# Immunohistochemical assessment of symptomatic postmenopausal endometrial polyps in tamoxifen users and nonusers: a case control study

**DOI:** 10.1590/1516-3180.2018.0346.R4.19112019

**Published:** 2020-03-23

**Authors:** Raquel Papandreus Dibi, Claudio Galleano Zettler, Carla Maria de Martini Vanin, Rafaela Vanin Pinto Ribeiro, Júlia Monteiro de Oliveira, Thaysa Guglieri Kremer, Josiane Borges, Sérgio Kakuta Kato

**Affiliations:** I MD, PhD. Professor, Department of Gynecology and Obstetrics, Universidade Federal de Ciências da Saúde de Porto Alegre (UFCSPA); and Professor, Gynecology and Obstetrics Service, Irmandade Santa Casa de Misericórdia de Porto Alegre (ISCMPA), Porto Alegre (RS), Brazil.; II MD, PhD. Pathologist, Pathology Service, Irmandade Santa Casa de Misericórdia de Porto Alegre (ISCMPA), Porto Alegre (RS), Brazil.; III MD, PhD. Professor, Department of Gynecology and Obstetrics, Universidade Federal de Ciências da Saúde de Porto Alegre (UFCSPA); and Professor, Gynecology and Obstetrics Service, Irmandade Santa Casa de Misericórdia de Porto Alegre (ISCMPA), Porto Alegre (RS), Brazil.; IV MD. Undergraduate Student, Universidade Federal do Rio Grande do Sul (UFRGS), Porto Alegre (RS), Brazil.; V Undergraduate Medical Student, Universidade Federal do Rio Grande do Sul (UFRGS), Porto Alegre (RS), Brazil.; VI Undergraduate Medical Student, Pontifícia Universidade Católica do Rio Grande do Sul (PUCRS), Porto Alegre (RS), Brazil.; VII MD. Pathologist, Pathology Service, Irmandade Santa Casa de Misericórdia de Porto Alegre (ISCMPA), Porto Alegre (RS), Brazil.; VIII PhD. Epidemiologist, Postgraduate Program on Pathology, Universidade Federal de Ciências da Saúde de Porto Alegre (UFCSPA), Porto Alegre (RS), Brazil.

**Keywords:** Polyps, Tamoxifen, Menopause, Immunohistochemistry, Gynecology, Endometrium, Endometrial polyps, Postmenopause, Hormone therapy

## Abstract

**BACKGROUND:**

Endometrial polyps are common in postmenopausal women, and the effect of tamoxifen use (a risk factor for endometrial polyps) on their pathogenesis is unclear.

**OBJECTIVES:**

To evaluate the expression of hormone receptors and markers for proliferation/apoptosis (Ki-67 and Bcl-2) in endometrial polyps in postmenopausal users and nonusers of tamoxifen.

**DESIGN AND SETTING:**

Cross-sectional analytical study in a tertiary-level academic hospital.

**METHODS:**

46 women (14 tamoxifen users and 32 nonusers) with postmenopausal bleeding underwent hysteroscopic resection of endometrial polyps. Polyp samples were immunohistochemically assessed for detection of Ki-67, Bcl-2 and estrogen and progesterone receptors.

**RESULTS:**

Analysis on the glandular component of the polyps revealed progesterone receptor expression in the polyps of 96.9% of the nonusers of tamoxifen, and 92.3% of the tamoxifen users (P = 0.499). All polyps in nonusers and 92.3% of those in users were also positive for estrogen receptors (P = 0.295). Ki-67 was expressed in 75% of the polyps in the tamoxifen users and 82.8% of those in the nonusers. All endometrial polyps expressed Bcl-2.

**CONCLUSIONS:**

The immunohistochemical analysis on endometrial polyps demonstrated that, although tamoxifen is considered to be a risk factor for endometrial polyps, there were no significant differences in the expression of hormone receptors between users and nonusers of tamoxifen. There were no between-group differences in Ki-67 and Bcl-2 expression, and all patients displayed inhibition of apoptosis by Bcl-2, thus supporting the theory that polyps develop due to inhibition of apoptosis, and not through cell proliferation.

## INTRODUCTION

Even though endometrial polyps are common in postmenopausal women, their pathogenesis is still not entirely understood.^1^ Polyps are most commonly diagnosed in the fifth decade of life,^2^ and occur in 16 to 54% of women with postmenopausal bleeding.^3^^,^^4^

Late menopause, hormone therapy and tamoxifen use are considered to be risk factors for endometrial polyps. Although the etiopathogenesis of these polyps is still unknown, their presence is considered to be a risk factor for endometrial cancer. Tamoxifen users have higher incidence of polyps, possibly due to the estrogenic effects of tamoxifen on the endometrial epithelium.^5^

## OBJECTIVE

Since there is no consensus in the literature regarding the pathogenesis of postmenopausal endometrial polyps, or regarding the effect of tamoxifen on the hormone receptor profile of these polyps, the aim of the present study was to investigate these issues. It was also sought to elucidate the effect of tamoxifen on markers for cell proliferation and apoptosis in postmenopausal polyps, given that the data in the literature are not consistent on this point.

## METHODS

### Patients

A cross-sectional analytical study was conducted on symptomatic postmenopausal women who were referred to the gynecological endoscopy service of the Santa Casa Hospital Complex for hysteroscopic examination between January 2000 and December 2003, and who underwent surgical hysteroscopy for polypectomy. These patients were recruited through convenience sampling and were classified into two groups according to their use of tamoxifen: group 1, nonusers (controls); and group 2, users (cases).

A total of 46 postmenopausal women aged between 47 and 86 years participated in the study. All of the patients presented postmenopausal bleeding and underwent hysteroscopic resection of endometrial polyps.

Fourteen patients had been using tamoxifen for at least one year to treat breast cancer (group 2), while the remaining patients had never used hormone medication (group 1). The length of time since the menopause ranged from one to 35 years.

The polyp samples were cut and mounted on slides (total of 184 slides). However, three slides containing estrogen and progesterone receptors, thirteen Ki-67 slides and four Bcl-2 slides were discarded due to difficulties in slide preparation and reading.

### Ethical considerations

This study was approved by our institution’s research ethics committee (institutional review board-equivalent) under the number 251.296, on April 22, 2013. The study was conducted in accordance with the provisions of the Declaration of Helsinki.

### Method

The immunohistochemical analysis was conducted using formalin-fixed and paraffin-embedded tumor tissue. The samples were then sectioned into slices of thickness 3 µm, deparaffinized and rehydrated prior to analysis. The Advance^ΤΜ^ HPR kit (DakoCytomation^®^) was used for detecting Ki-67, Bcl-2 and estrogen and progesterone receptors.

Sodium citrate (pH 6.0) was used for Ki-67 antigen retrieval. Antigenic retrieval of Bcl-2, estrogen and progesterone was conducted using Tris-ethylenediaminetetraacetic acid (EDTA) (pH 9.0), through incubation at 95-98 °C in a water bath for 40 minutes. Endogenous peroxidase activity was blocked through incubation in 5% hydrogen peroxide (H_2_O_2_) 30V in methanol for two ten-minute periods. Nonspecific proteins were blocked using 1% bovine serum albumin for 30 minutes.

The slides were incubated with primary antibodies overnight at 4 °C. They were also subjected to incubation with secondary and tertiary antibodies for 40 minutes at room temperature. Samples of lymphoid tissue (tonsil samples) were used as a positive control for Ki-67 and Bcl-2, and mammary tissue was used as a control for the analysis on estrogen and progesterone. The same tissues were also incubated with all but the primary antibodies, which were replaced with 1% bovine serum albumin, as a form of negative control.

Antibody-antigen binding was viewed using diaminobenzidine chromogen. Harris hematoxylin was used for counterstaining, and the slides were dehydrated and mounted with synthetic resin.

All the immunostained slides for each antibody were analyzed and quantified separately by two observers using an optical microscope. Immunopositivity for estrogen, progesterone, Ki-67 and Bcl-2 was investigated in at least 10 high-power fields, and the results were considered based on immunoreactivity in the glandular epithelium.

Cases were considered positive for estrogen and progesterone receptors when immunostaining was observed in at least 1% of the gland cells in the sample, and were considered negative when immunostaining was not observed, or occurred in less than 1% of the sample.

The analysis on Bcl-2 was conducted through cytoplasmic immunostaining of gland cells. Absence of staining in the endometrial gland was indicative of a negative result, and reactions in at least one endometrial gland were considered to be positive results.

Ki-67 immunoreactivity was semi-quantitatively analyzed through nuclear immunostaining in the glandular component in the samples. Immunostaining in at least 5% of gland cell nuclei was considered indicative of high proliferation. Cases with an absence of immunostaining or immunoreactivity in less than 5% of cells were suggestive of low proliferation indices.

### Statistical analysis

Qualitative variables were described as absolute and relative frequencies, and quantitative variables as the mean and standard deviation or the median and interquartile range.

Between-group comparisons of mean patient age and menopausal age were conducted using a t-test. Fisher’s exact test was used to investigate associations between the presence or absence of hormone receptors (estrogen and progesterone) and markers for proliferation and apoptosis (Ki-67 and Bcl-2). The Mann-Whitney nonparametric test was used for between-group comparisons of the length of time since menopause and the presence or absence of progesterone receptors.

The analyses were performed using the Statistical Package for the Social Sciences (SPSS) software, version 17.0, and a 5% significance level was used.

## RESULTS

The results regarding the expression of estrogen and progesterone receptors and proliferation (Ki-67) and apoptosis (Bcl-2) markers in the endometrial polyps of postmenopausal users (group 2) and nonusers of tamoxifen (group 1) are displayed in Table 1.

**Table 1.** Prevalence of hormone receptors (ER and PR) and markers for proliferation and apoptosis (Ki-67 and Bcl-2), according to tamoxifen use

Positive	Tamoxifen users Group 1	Tamoxifen nonusers Group 2	P
PR	31 (96.9%)	12 (92.3%)	0.499
ER	31 (100.0%)	12 (92.3%)	0.295
KI-67	24 (82.8%)	9 (75.0%)	0.627
Bcl-2	30 (100.0)	12 (100.0)	-

PR = progesterone receptor; ER = estrogen receptor.

Estrogen and progesterone receptors were identified in the glandular tissue of endometrial polyps in both groups, and no between-group differences were found in relation to this variable. The mean age of the patients in group 1 was 60.91 years, while the mean age in group 2 was 63.87 years, with no significant difference between the groups (P = 0.360).

As shown in Table 2, the mean age at which menopause occurred did not differ between the users (M = 49.29 years) and nonusers (M = 49.90) of tamoxifen (P = 0.727). Analysis on the length of time since the menopause and hormone receptor expression showed that the length of time since the menopause was significantly greater in the absence (median of 29 years) than in the presence (median of 9 years) of progesterone receptors, as shown in Table 3 (P = 0.026).

**Table 2.** Sample characteristics

	Tamoxifen users	Tamoxifen nonusers	P
	Group 1	Group 2	
Age	60.91 ± 10.08	63.86 ± 9.66	0.360
Age at menopause	49.90 ± 4.02	49.29 ± 5.86	0.727
Length of time since menopause (years)	8.00 (3.75-14.00)	10.50 (7.00-21.75)	0.161

Mean ± standard deviation; median (P25-P75).

**Table 3.** Median and interquartile range of length of time since the menopause, according to presence or absence of progesterone receptors

	Progesterone receptors		P
	Positive	Negative	
Length of time since menopause	9.00 (3.50-12.50)	28.00 (12.25-34.00)	0.026

All the patients had initially reported experiencing postmenopausal bleeding, and after ultrasound findings suggestive of endometrial polyps, all of them underwent hysteroscopy. The median length of time since the menopause at the time of hysteroscopic resection of endometrial polyps was 10.50 years (interquartile range 7.00-21.75) among users of tamoxifen and 8.00 years (interquartile range 3.76-14.00) among nonusers (P = 0.161). An inverse correlation was identified between the length of time since the menopause and the expression of progesterone receptors, as displayed in Table 3 (P = 0.026).

After hysteroscopic resection, the polyps were classified as hyperplastic or atrophic. Atypical hyperplasia and cancer were not observed in any of the cases examined.

## DISCUSSION

In the present sample, the similarities in mean patient age and age at the menopause between the groups were indicative of a homogenous and representative sample.

The pathogenesis of endometrial polyps is still not well understood, and there is no consensus regarding the treatment of postmenopausal polyps. Biron-Shental et al.^6^ identified endometrial polyps in 25.9% of a sample of postmenopausal breast cancer patients who used tamoxifen. It has been reported that polyps become malignant in 3-10.7% of the cases.^7^^,^^8^^,^^9^

The role of estrogen in the development of endometrial polyps can be explored by assessing hormone receptors. Studies have shown that a reduced number of progestogen receptors (compared with estrogen receptors) are present in the stromal but not the glandular component of polyps.^10^^,^^11^ In the present sample, glandular expression of progesterone receptors was found in 92.3% of the users of tamoxifen and 96.9% of the nonusers (P = 0.499). A total of 92.3% of the polyps in the tamoxifen users were estrogen-positive, while all the polyps of the nonusers displayed estrogen (P = 0.295).

These results demonstrate that, in the present sample, tamoxifen use did not significantly influence the expression of progesterone and estrogen receptors in postmenopausal endometrial polyps. Endometrial polyps may develop due to increased expression of estrogen receptors, reduced expression of progesterone receptors, or both.^10^ In the present sample, no significant difference between the expressions of estrogen and progesterone receptors was found in endometrial polyps.

Taylor et al.^11^ found that the increased expression of estrogen in polyps was limited to the glandular epithelium. Although the analyses in the present study were limited to the glandular epithelium, the findings are in agreement with those of Taylor et al.^11^

Studies on the pathogenesis of endometrial polyps have suggested that an imbalance between estrogen and progesterone may play a role in polyp growth.^3^^,^^10^^,^^11^^,^^12^^,^^13^^,^^14^^,^^15^ Some studies have demonstrated decreased expression and others increased expression of hormone receptors in polyps.

Almeida et al.^3^ assessed hormone receptors in endometrial polyps and the surrounding endometrium in postmenopausal women and found higher expression of estrogen and progesterone receptors in the polyps. They therefore suggested that low estrogen levels and endometrial atrophy might contribute to polyp development. They concluded that hormone receptors, especially estrogen, played an important role in the physiopathology of postmenopausal endometrial polyps.

Studies such as those by Mittal et al.^10^ and Belisario et al.^16^ have suggested that, in the absence of high estrogen levels, increased expression of estrogen and progesterone receptors in gland cells may contribute to polyp development. These results suggest that the expression of estrogen and progesterone receptors differs between the stromal and glandular endometrium in postmenopausal patients.

Koshiyama et al.^12^ studied 33 menopausal patients and found that there was a reduction in estrogen level with increasing age. In the present sample, the reductions in the numbers of hormone receptors were also proportional to the length of time since the menopause (P = 0.026).

It was found in the present study that the polyps in 75% of the tamoxifen users and 82.8% of the nonusers expressed Ki-67. The present results are in agreement with the data in the current literature, in that no between-group differences were found regarding Ki-67 expression.^17^

During the normal menstrual cycle, endometrial apoptosis occurs in the middle and at the end of the secretory phase, and during the first two days of menstruation. The balance between mitotic activity and apoptosis regulates endometrial development during the menstrual cycle.^18^ The expression of Bcl-2 genes is inversely proportional to the level of apoptosis in the tissue. In the present sample, Bcl-2 was expressed in the endometrial polyps of all patients (users and nonusers of tamoxifen).

Morsi et al.^19^ identified significant Bcl-2 expression in the glandular component of the endometrium in postmenopausal women, despite minimal expression in the endometrial stroma. Some studies have suggested that endometrial polyp growth occurs due to decreased apoptosis, characterized by increased Bcl-2 expression.^11^^,^^20^

One potential source of bias in the present study was that the participants were recruited through convenience sampling among women living in southern Brazil who presented symptomatic postmenopausal bleeding and were referred to a tertiary medical center for investigation. The best information regarding patients who underwent hysteroscopy was found to be over the period from 2000 to 2003. The sample of consecutive patients was selected according to the patients’ records, and then immunohistochemical evaluation was performed. No computer system was used to analyze the immunohistochemical technique.

Despite these limitations, the present study provides an analysis on several consecutive cases and controls from a specific population of postmenopausal women who were either users or nonusers of tamoxifen and presented symptomatic endometrial polyps that were resected and immunohistochemically assessed. It is essential to emphasize the relevance of conducting immunohistochemical studies among patients who are using tamoxifen and of ascertaining the pathogenesis of the endometrial disease in this specific population, along with the role of hormone receptors in the pathogenesis of endometrial polyps. Although the study sample was small, this is a very important subject. Therefore, we would encourage further studies with the aim of clarifying the study questions.

## CONCLUSIONS

Longer time since the menopause was associated with decreased expression of hormone receptors in endometrial polyps. Immunohistochemical analyses showed that, although tamoxifen use is generally considered a risk factor for developing endometrial polyps, hormone receptor expression did not differ between users and nonusers of tamoxifen. No between-group differences in Ki-67 and Bcl-2 expression were observed, and inhibition of apoptosis through Bcl-2 overexpression was observed in all participants. These findings support the theory that polyp growth occurs due to inhibition of apoptosis, rather than because of cell proliferation alone.

In the functional endometrium, apoptosis is related to hormone receptor expression and varies over the menstrual cycle. In postmenopausal women, apoptosis does not appear to be regulated by estrogen and progesterone.
